# In Situ Thermosensitive Mucoadhesive Nasal Gel Containing Sumatriptan: *In Vitro* and *Ex Vivo* Evaluations

**DOI:** 10.3390/polym16233422

**Published:** 2024-12-05

**Authors:** Aseel Alshraim, Doaa Alshora, Lubna Ashri, Ahlam Alhusaini, Nawal Alanazi, Nisreen M. Safwan

**Affiliations:** 1 Department of Pharmaceutics, College of Pharmacy, King Saud University, Riyadh 11451, Saudi Arabia; aseel.alshraim@gmail.com (A.A.); lashri@ksu.edu.sa (L.A.); nalanaze@ksu.edu.sa (N.A.); 2 Department of Pharmacology, College of Pharmacy, King Saud University, Riyadh 11451, Saudi Arabia; aelhusaini@ksu.edu.sa; 3 College of Pharmacy, King Saud University, Riyadh 11451, Saudi Arabia; nalnakeli@ksu.edu.sa

**Keywords:** sumatriptan, thermo-responsive, *in situ* gel, nasal drug delivery

## Abstract

The aim of this study was to develop a thermosensitive mucoadhesive (MA) in situ nasal gel for sumatriptan. A 3D response surface methodology (Design of Expert version 11) was employed to formulate nine different formulations. The Pluronic F-127 concentration (X1) and chitosan concentration (X2) were selected as independent factors. The formulas were studied in terms of pH, clarity, drug content, gelation temperature, gelation time, gel strength, MA strength, viscosity, % release after 5 h, and release kinetics. The optimized formulas were studied for % permeated after 5 h and stability in addition to previous tests. The study of the stability of the optimized formula was performed under accelerated conditions (40 ± 2 °C, 75 ± 5% RH) for 3 months. The outcomes of the optimized formula were a clear gel with a gelation temperature of 33 °C and a reasonable gelation time of less than one minute, and the release and permeation during 5 h were 40% and 50%, respectively. The formulated gel decreased the mucociliary clearance (MCC) and thus increased the retention time in the nasal cavity, resulting in enhancing SMT absorption, which could improve the drug efficacy.

## 1. Introduction

A migraine, which is pain in one-half of the head, is one of the most common multisymptomatic disorders, which currently and unfortunately has no cure, and its therapy management includes acute symptomatic relief and episode prevention [1,2]. Triptans are among the most important agents for the treatment of migraines, with sumatriptan (SMT) being the first and the most commonly prescribed drug of them. It was initially introduced as a subcutaneous injection providing fast and complete relief of symptomatic migraines, yet had high incidence of adverse events and poor patient compliance. Afterward, oral SMT was introduced as more convenient route but was associated with gastrointestinal (GI) incompatibility, low absolute bioavailability, and a short half-life [2].

Along with the mentioned routes, the nasal route for SMT, which is a spray, is an alternative for the oral route since it can avoid the limitations and improve the speed and consistency of drug absorption. However, it is associated with limitations, which are a short retention time, due to the mucociliary clearance (MC) and a dual form of absorption (nasal and GI); thus, a portion of the dose will be subjected to first-pass metabolism, leading to low bioavailability and taste disturbance [2,3,4].

Nasal *in situ* gel delivery systems, where many drugs are successfully formulated and delivered, are shown to be promising formulations due to their ability to overcome the marketed SMT nasal spray limitations [5].

Many studies were conducted to develop nasal *in situ* gel delivery systems for SMT that appeared to be effective and safe for SMT delivery. Pluronic and carbopol polymers were used, and the formulation had an increased SMT absorption [6]. In addition, a blend of polymers was successfully incorporated in an SMT *in situ* gel, resulting in a stable and sustained drug release [7]. Furthermore, a formulation developed using Pluronic and HPMC successfully increased SMT bioavailability [8]. Pluronic and hyaluronic acid polymers gave rise to prolonged residence time and enhanced permeation when used in SMT *in situ* gel [9].

Pluronic is an essential thermoresponsive polymer with numerous types, and Plu-127 is one of the most popular types, owing to its ability to form a gel at a comparatively low concentration at body temperature. However, its rapid erosion, weak bioadhesion, and low gelation temperature necessitate its use with other polymers like chitosan, HPMC, and carbopol. Chitosan is a cationic, naturally occurring linear polysaccharide extracted from crustacean shells. It is a thermoresponsive polymer commonly used as a drug delivery vehicle in the pharmaceutical industry. Its positive charge can easily interact with the negatively charged nasal mucosa. Also, it significantly enhances nasal permeation and absorption of the drug. It is a biodegradable, biocompatible polymer with excellent mucoadhesive behavior, which promotes paracellular drug transport and increases nasal retention time. These chitosan-based gel features make it a promising candidate for nasal drug delivery [10,11,12].

The study aimed to develop, optimize, and characterize a thermosensitive mucoadhesive *in situ* nasal gel of SMT using Plu F-127 and chitosan polymers using a full factorial design (3^2^). The prepared *in situ* gel offers advantages over the marketed SMT nasal spray by increasing the bioavailability and avoiding taste disturbance with a fast onset of action. In addition, it offers advantages over other SMT in situ gels since chitosan has an excellent MA behavior, which increases the nasal retention time of the formulation and may cross the blood-brain barrier.

## 2. Material and Methods

### 2.1. Materials

SMT was gifted from Tabuk Pharmaceuticals, (Tabuk, Saudi Arabia). Plu F-127 (CAS. No: 9003-11-6) and chitosan with high molecular weight (HMW) (75% deacetylated) (CAS. No: 9012-76-4) were purchased from Sigma-Aldrich Company (Schnelldorf, Germany). Calcium chloride was obtained from Romil Company (Cambridge, UK), potassium chloride was obtained from BHD Company (London, UK), and sodium chloride and triethanolamine were obtained from Loba Chemie Company (Mumbai, India).

### 2.2. Methods

#### 2.2.1. Formulation of SMT-Loaded Nasal In Situ Gel Systems

The nasal *in situ* gel formulations were prepared using the cold method [13]. Different concentrations of Plu F-127 were dissolved in cold distilled water (4 °C) and were kept in the refrigerator for 24 h for the complete desolvation of all Plu F-127 particles. HMW chitosan with different concentrations was dissolved in 0.5% glacial acetic acid and kept overnight in a refrigerator. To prepare the different drug-containing gel formulations, SMT was dissolved in Plu F-127 solution with continuous stirring to obtain a homogenous clear solution of Plu F-127-SMT. Chitosan was then added to the solution at 4 °C at a ratio of 9:1 Plu F-127 to chitosan.

#### 2.2.2. Optimization of SMT In Situ Gel Formulation

The impact of two independent factors, the Plu-127 concentration (X1, at levels of 13.5, 18, and 22.5%) and the chitosan concentration (X2, at levels of 0.01, 0.03, and 0.05%), on different attributes was studied using 32 full factorial designs (Design-Expert 11). These attributes were gelation temperature (Y1), gelation time (Y2), MA strength (Y3), and *in vitro* release (Y4). Table 1 represents the composition of each formulation.

#### 2.2.3. Characterization of Gel

##### Clarity and pH of the Formulations

The clarity of all formulations was determined visually. They were checked against a white and black background and graded as turbid, clear, and very clear. The pH of each formulation was determined by using a pH meter, considering that nasal formulations should have a pH ranging between 5.5 and 6.5 [14].

##### Gelation Temperature

The gelation temperature was determined by placing 2 mL of the refrigerated formula in a 10 mL test tube with a diameter of 1.0 cm and closed using parafilm. The tube was placed in a water bath at a temperature of 4 °C, and the temperature was incremented slowly to 5 °C at the beginning of the experiment until it reached 20 °C, then 1 °C when the temperature reached the region of the sol–gel transition temperature. Equilibration was allowed for 10 min after each temperature increment until gelation occurred. The test tube was tilted at a 90° angle to confirm gelation had occurred and that the meniscus of the preparation did not move during slanting, and then the gelation temperature was recorded [15,16].

##### Gelation Time

One gram of the prepared gel was added to 10 mL of simulated nasal fluid (SNF) composed of 8.77 g NaCl, 2.98 g KCl, 0.59 g CaCl_2_, and distilled water up to 1000 mL. The gelling time was visually observed and graded according to the time needed to form the gel [15,16].

##### Gel Strength

The gel strength was determined to indicate the viscosity of the nasal *in situ* gel at physiological conditions. It was the time (seconds) required by a weight equal to 3.5 g to penetrate a 0.5 cm distance through a 5 g gel formulation placed in a 10 mL measuring cylinder [17,18].

##### Mucoadhesive Strength

The force required to detach the formulation from nasal mucosal tissue was determined. A modified balance technique was used with a beaker on one side of the balance, and on the other side, a vial was fitted with sheep nasal mucosa (with a thickness of 0.6 mm cut from the sheep nose mucosa obtained from slaughtered sheep). The nasal mucosa was fitted on the bottom of the vial. The vials were kept at 32–34 °C for 10 min. Then, 1 mL of the gel sample was placed in a watch glass underneath the vial fitted with the nasal tissue. The vial was then pressed to the gel sample for one minute as the initial contact time. Then, water was slowly added to the beaker on the other side of the balance using a pipette [18].

(1) Mucoadhesive strength=m×g/A 

where m is the weight required for detachment in grams, g is the acceleration due to gravity (980 cm/s^2^), and A is the area of mucosa exposed.

##### Viscosity

The viscosity of all formulations was measured using a Brookfield viscometer (Manchester, UK) with a small volume adapter. The temperature sensing probe was lowered into the gel, and the gel’s temperature was recorded. The viscosity at 32 °C temperatures was denoted [19].

#### 2.2.4. Drug Content

Accurately, 1 g of the gel formulation was diluted to 10 mL with SNF, and then 1 mL of this solution was diluted to 10 mL with SNF. The absorbance of the prepared solution was measured spectrophotomically at 283 nm [19].

#### 2.2.5. *In Vitro* Drug Release

*In vitro* drug diffusion study of all formulations was performed using a Franz diffusion cell. The dialysis membrane (molecular weight cutoff range of 12–14 kDa) was soaked in SNF for 24 h before the experiment. Diffusion cells were filled with 21 mL SNF, and the dialysis membrane was mounted on the cells. One (1) gram of gel was placed into the donor chamber, and the temperature was maintained at 34 °C by a shaker water bath at 50 rpm. Samples of 1 mL were withdrawn at different time intervals, replaced with the same volume of fresh solution, and filtered. The amount of drug was determined by an ultraviolet (UV) visible spectrophotometer at 283 nm [19].

#### 2.2.6. Release Kinetics

The data were fitted to different models, including the following:

(a) Zero-order

(2) $$Q_{t}=Q_{0}+k_{0}\;t$$

where Q_t_ is the amount of a drug released at time t, Q_0_ is the initial amount of drug, and k_0_ is the zero-order release rate constant. The percentage cumulative amount of a drug released was plotted versus time [20,21].

(b) First-order

Here, the log percentage cumulative of remaining drug was plotted against the time, and a straight line with a slope equal to K/2.303 was obtained.

(3) $$Log\;Q_{t}={\log Q}_{0}-\frac{kt}{2.303}$$

where k is the first-order release rate constant [19,20].

(c) Higuchi model

The cumulative release percent was plotted versus the square root of time.

(4) $$Q_{t}=kt^{1/2}$$

where k is the Higuchi’s release rate constant [19,21].

(d) Korsmeyer–Peppas model

In this model, the log of percent cumulative release data was plotted against the log time.

(5) $$Q_{t}/Q_{\infty }=kt^{n}$$

where Qt/Q∞ is the fraction of drug release at time t, k is the kinetic constant, and n is the release exponent, which depends on the release mechanism. Thus, it is used to characterize it as either Fickian diffusion (n ≤ 0.45) or anomalous diffusion (non-Fickian transport), where 0.45 < n < 0.89, n = 0.89 indicates case II (relaxational) transport, and n > 0.89 indicates super case II transport [22,23].

#### 2.2.7. DSC Characterization

Thermograms of the SMT-optimized formulation compared with the untreated SMT and the individual components of the in situ gel, as well as the physical mixture, were recorded by DSC for thermal analysis using the DS calorimeter DSC 8000 Perkins Elmer (Waltham, MA, USA). The samples (3–5 mg) were sealed in aluminum pans and heated at a constant rate of 10 °C/min, over a temperature range of 30 °C to 350 °C. Thermograms of the samples were then obtained using a software program in a TA50I PC system.

#### 2.2.8. Fourier Transform Infrared Spectroscopy (FTIR)

The FTIR spectra for the SMT, chitosan, and Plu F-127, as well as their physical mixture, were investigated. The substances were identified based on spectral analysis over a wavenumber range from 4000 to 500 cm^−1^; potassium bromide (spectroscopic grade) was mixed with the samples and compressed into disks using a hydraulic press. The data were analyzed using the Perkin Elmer (Spectrum V5.3.1) program.

#### 2.2.9. *Ex Vivo* Permeation Studies

Fresh nasal mucosa from the olfactory region was carefully removed from the nasal cavity of sheep obtained from the local slaughterhouse. The nasal mucosa was inserted into nasal saline buffer, pH 6.4. Tissue samples were placed on diffusion cells immediately. One (1) gram of gel containing the optimized formula was placed onto the donor chamber of the Franz cell diffusion apparatus. The temperature was maintained at 34 °C by circulating a water bath. Samples of 1 mL were withdrawn at different time intervals, replaced with the same volume of fresh solution, and filtered. A UV-visible spectrophotometer determined the amount of drug at 283 nm. The permeation parameters for SMT, the steady state fluxes (Jss), and permeability coefficients (P) were calculated from the *ex vivo* permeation data across sheep nasal membranes [19,24].

#### 2.2.10. Stability Study

The stability of the optimized formula was monitored for 3 months at 40 ± 2 °C, 75 ± 5% RH. The determination was in terms of the gelation temperature, pH, viscosity, drug content, and drug release [25]. The degradation rate constant (K) was calculated by plotting the logarithms of the percent of the drug remaining against the time (days), and the slope was equal to K/2.303.

#### 2.2.11. Statistical Analysis

Data were presented as mean and standard deviation (SD). A one-way analysis of variance (ANOVA) followed by post hoc analysis was used to compare the data. A *p*-value < 0.05 was considered to indicate a significant difference.

## 3. Results and Discussion

### 3.1. Gel Characterization

Table 2 shows the characterization of different SMT in situ gel. All formulations were clear and had a pH in the acceptable range (5.5–6.5). In addition, the drug content was in the range 85–115%.

### 3.2. Rheological Studies

Table 3 shows the rheological pattern of SMT gel formulations. Noticeably, all formulations remained liquid at room temperature. The transition to gel-like behavior took place with increased temperature. Adding or increasing the shear stress from 20 to 100 rpm increased the viscosity, resulting in a shear-thickening behavior. Moreover, it was clear that the concentration of Plu F127 directly affected the viscosity, which could be attributed to a sudden increase in micellar concentration at higher temperatures. The profile of viscosity followed a shear-thickening pattern indicating sol-to-gel conversion [26,27]. The nasal in situ gel should have a low viscosity at room temperature, allowing it to be administered. After that, transforming to gel and higher viscosity may decrease the clearance from the nasal cavity and increase the intact time with nasal mucosa.

### 3.3. Effect of Independent Factors

#### 3.3.1. Effect of Independent Factors on Gelation Time

ANOVA table analysis showed the significance of the independent parameter on the gelation time (*p* < 0.0001). It was found that the concentration of PLU F-127 and its quadratic effect had a significant effect on the gelation temperature. In contrast, the chitosan concentration, its quadratic effect, and the interactive effect were insignificant.

**Figure 1:**
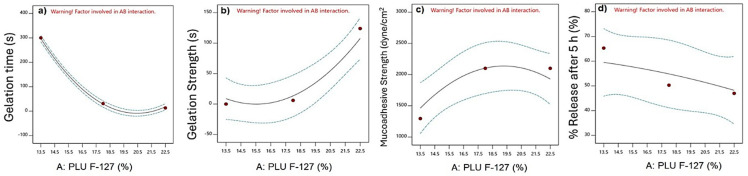
Effect of Pluronic F-127 on the gelation temperature (**a**), gelation strength (**b**), mucoadhesive strength (**c**), and % release after 5 h (**d**).

**Figure 2:**
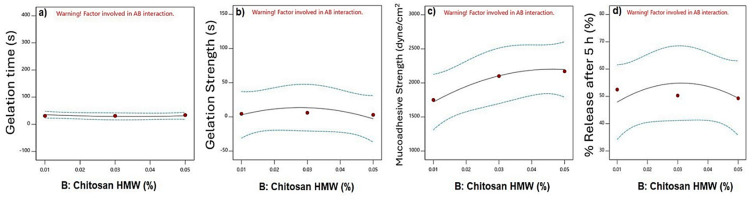
Effect of chitosan HMW on the gelation temperature (**a**), gelation strength (**b**), mucoadhesive strength (**c**), and % release after 5 h (**d**).

**Figure 3:**
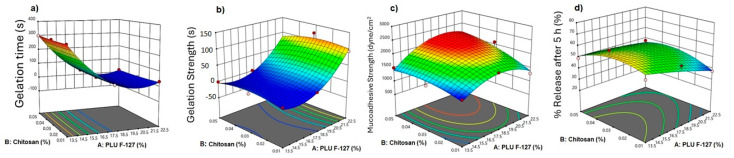
A 3D response plot for the gelation temperature (**a**), gelation strength (**b**), mucoadhesive strength (**c**), and % release after 5 h (**d**).

Figure 1a shows the effect of PLU F-127 on the gelation time. Increasing the PLU F-127 concentration from 13.5 to 22.5%, as in F1 and F3, respectively, showed a significant reduction in the gelation time from 300 to 32 s. Figure 2a shows that chitosan had no effect on the gelation time. The 3D response surface plot is represented in Figure 3a. The gelation time ranged from 300 to 13 s. A significant reduction in the gelation time was observed when the PLU concentration was increased.

The same finding was observed by Morsi and her colleagues, who found that the gelation time of the in situ gel containing Ketorolac tromethamine decreased with increasing PLU F-127 concentration [28]. Also, Unal and his colleagues had the same results when formulating a sustained release in situ gel loaded with paclitaxel, and it was found that increasing the PLU F-127 concentration decreased the gelation time and temperature owing to its aptitude to form a gel at a comparatively low concentration at body temperature [29]. The exact mechanism of gelation is not adequately known, although studies suggest that gelation occurs due to changes in intrinsic micellar behavior and entanglement of micelles at higher temperatures. The rising temperature changes the methyl group orientation in the side chain and causes dehydration of the polypropylene oxide block. In addition, it causes the expulsion of water from the micelle core. All of this results in the gelation of the aqueous solution of poloxamer [10].

#### 3.3.2. Effect on Gelation Strength

The ANOVA analysis showed that the influence of PLU-F127 and its quadratic effect on gelation strength was significant (*p* < 0.0001). On the other hand, chitosan concentration and its quadratic effect and the interactive effect are shown in the table to be insignificant. The results revealed that the gelation strength increased significantly from 0.003 s to 94 s by increasing the PLU F-127 concentration from 13.5% to 22.5% (Figure 1b) and no pronounced effect of chitosan (Figure 2b). The gelation strength increased from 0.003 s to more than 100 s by increasing the PLU-F127 concentration as shown in the 3D response surface plot (Figure 2b). Hussein and his colleagues had the same direct relationship. However, they used hyaluronic acid instead of chitosan to create SMT in situ nasal gel [9]. In addition, Godbole and his colleagues, who used PLU-F127, PLU 188, and HPMC K4M to develop an in situ nasal gel for Zolmitriptan, also found the same direct relationship. They suggested that this relationship might be related to hydrogen bonding between PLU-F127 and bioadhesive polymers in the nasal gel [30].

#### 3.3.3. Effect on Mucoadhesive Strength

The analysis showed that the individual effects of PLU F-127 and chitosan and the quadratic effect of PLU F-127 had a significant impact, with *p* values < 0.05. The results showed a slight increase in MA strength when the chitosan concentration increased as in F2, F5, and F8, which had chitosan concentrations of 0.01%, 0.03%, and 0.05%, respectively (Figure 2c). From the individual effect plot (Figure 1c), increasing the PLU-F-127 concentration from 13.5 to 18.5% increased the MA strength as in F7 and F8, where the MA strength rose from 1505 to 2170. Increasing the PLU F-127 to 22.5% caused a plateau or slight decrease in the MA strength. Moreover, chitosan affected the response in the same way as PLU F-127, increasing the chitosan concentration from 0.01 to 0.03% as in F2, F5, and F8. The 3D response surface plot (Figure 3c), which showed the highest MA strength, resulted in a high concentration of PLU F-127 and chitosan as in F8 and F9. These results indicated that the variation in chitosan concentration showed changes in MA strength. A slight increase was observed in MA strength as the level of chitosan increased. This was probably due to the positive charge of chitosan, which could easily interact with the negatively charged nasal mucosa. The stronger the MA force was, the more it could prevent the gelled solution from coming out of the nose [10].

#### 3.3.4. *In Vitro* Released

**Figure 4:**
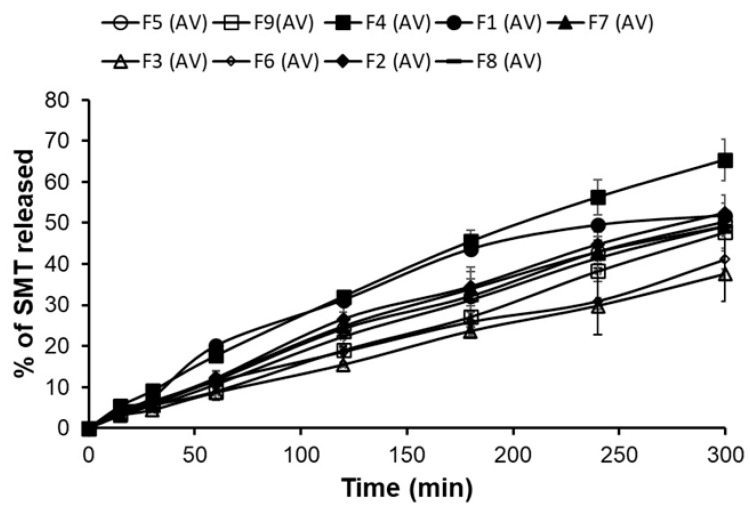
SMT releases from the in situ nasal gel for each formula over 5 h.

Figure 4 shows the release profile of the nine matrix *in situ* gels loaded with SMT. The ANOVA analysis showed no significant effect for PLU F-127 and chitosan concentrations nor their quadratic and interactive effects. However, the main plot in the Figure 3d shows that increasing the PLU F-127 concentration from 13.5 to 22.5% dramatically decreased the release from 65.349 to 41.109 (Figure 1d) as in F4 and F6, respectively, while increasing chitosan from 0.01 to 0.3 slightly increased the release, which was then reduced with further chitosan concentration increases (Figure 2d). The same observation was recognized by the 3D response plot (Figure 3d) as it was found that more sustained release was obtained with increasing PLU F-127. High PLU F-127 concentrations were related to decreased gelation time. Thus, a short gelation time was essential to prevent drainage from the application site and increase the residence time in the application area, resulting in prolonged release.

#### 3.3.5. Release Kinetics

Table 4 shows the release kinetics of SMT from different formulations. The release data for the zero-order, first-order, Higuchi, and Korsmeyer–Peppas models are plotted. The fitting of the release kinetics was determined using the highest correlation coefficient (r). The release kinetics data showed that all formulations fit the Higuchi model. To determine the mechanism of the release, the exponent n was calculated using the Korsmeyer–Peppas model. The *n* values were greater than 0.8, indicating a super case II transport and the drug released by diffusion and relaxation mechanisms.

### 3.4. Optimized Formula

The desirability of the optimum formula was based on low gelling time, maximum gel strength, mucoadhesive strength, and minimum release. Table 5 shows a comparison result between the predicted and observed values for the optimized formulation. The optimized formula transformed to gel at 33 ± 0.11 °C after 53 ± 1 s. The mucoadhesive strength was 1423 dyne/cm, and the % released after 5 h was 42.7 ± 1.42%. The release kinetics of the optimized formula best fit the Higuchi model with a regression coefficient of 0.994. The exponent n value calculated from Korsmeyer–Peppas equaled 0.86, an indication of case II relaxation in which the release of the drug depended on both diffusion and polymer chain relaxation.

### 3.5. DSC Characterization

**Figure 5:**
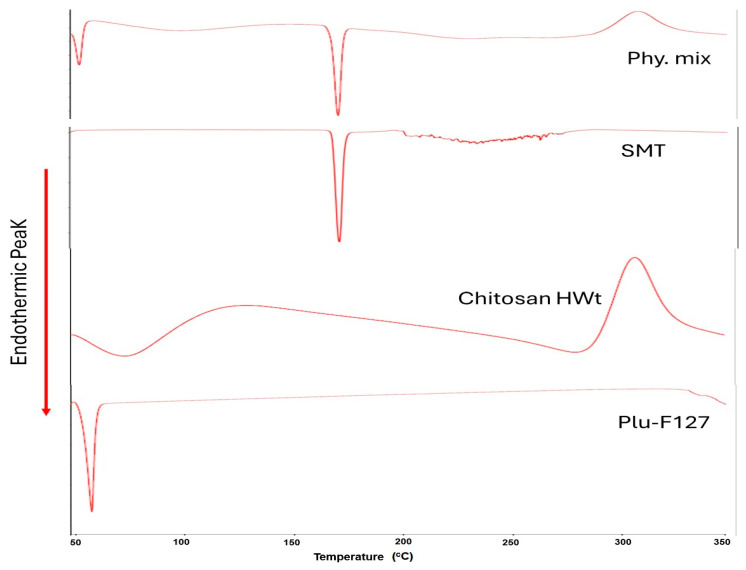
DSC thermograms for sumatriptan, chitosan, Pluromic F-127, and their physical mixture.

Figure 5 shows the DSC thermogram of the individual components and the physical mixture. Pluronic F-127 exhibited a sharp melting endotherm at 59 °C. Chitosan showed two peaks; an endothermic peak appeared at 65.9 °C, corresponding to the dehydration temperature, while the exothermic peak, which appeared at 307 °C, was related to chitosan decomposition. The DSC thermogram of sumatriptan succinate showed a sharp endothermic peak at nearly 171 °C, corresponding to its melting transition point. The DSC thermogram of the physical mixture shows all individual components peaks at the same position. This may reflect that no interaction occurred in the formulation.

### 3.6. Fourier Transform Infrared Spectroscopy (FTIR)

**Figure 6:**
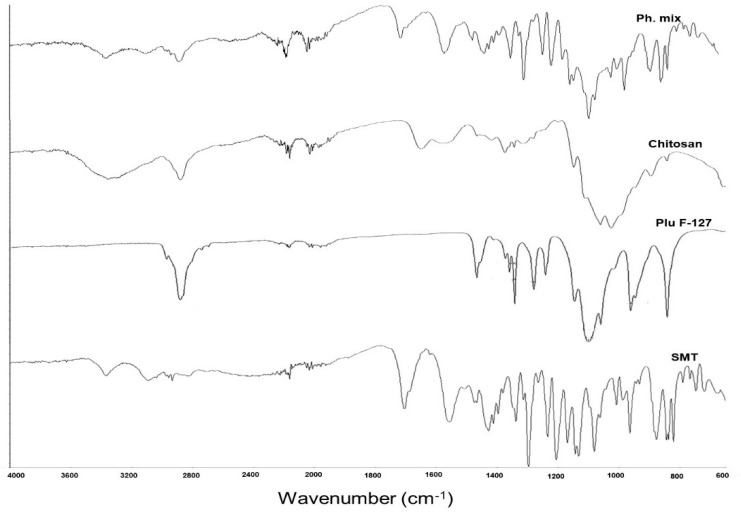
FTIR spectra for SMT, chitosan, Plu F-127, and their physical mixture.

Figure 6 shows the FTIR spectra for SMT, Plu f-127, CH, and their physical mixture. The spectrum of chitosan showed its characteristic signals, a broad absorption between 3500 and 3200 cm^−1^ attributed to the stretching vibration of O–H and N–H and another more discrete signal around 2879.22 cm^−1^ associated with the stretching vibration of CH. On the other hand, signals were observed at 1650.96 cm^−1^ attributed to the stretching vibration of C=O in the amide I group and at 1560 cm^−1^ attributed to the N–H bending and the C–N stretching vibrations in the amide II group. For Plu F-127, strong signals could be observed corresponding to the stretching vibrations of different bonds: 2880 cm^−1^ (C–H stretch), 1279.54 and 1241.45 cm^−1^ (C–O–C stretches), and 1100 cm^−1^ (C–O stretch). FTIR spectroscopic analysis of sumatriptan succinate showed the presence of the following peaks substantive of sumatriptan succinate: C–H stretching and bending, 1427.93, 877.35, and 821.69 cm^−1^; C–N stretching and bending, 1206.04 cm^−1^; C–O stretching, 1076.93, 1138.53, and 1204.08 cm^−1^ (alcoholic group); C=O, 1704.64 cm^−1^ (ketone group); C–H, 2933.86 cm^−1^; and C=C, 1558.64 cm^−1^. Thus, it confirmed the drug-excipient compatibility. The physical mixture kept the characteristic band of SMT.

### 3.7. Ex Vivo: %SMT Permeated

**Figure 7:**
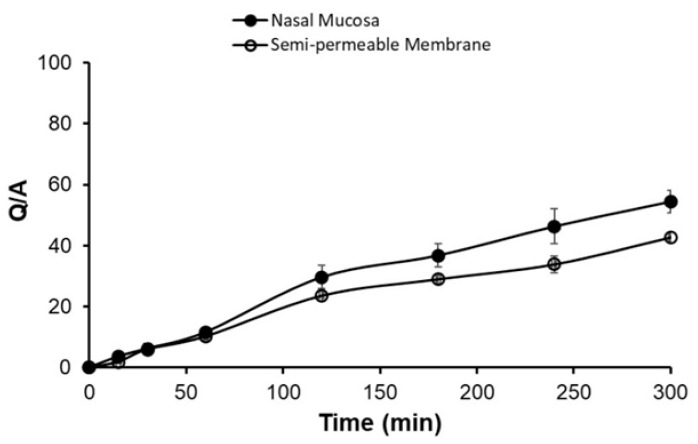
Cumulative amount of sumatriptan permeated.

*Ex vivo* permeation was observed for the optimized formulation using sheep olfactory nasal mucosa, which had a thickness of about 0.6 mm. It was observed that the permeation of the optimized formulation showed a release of 54.51 ± 3.6% at the end of 300 min, as shown in Figure 7. The flux was 90.86 µg/cm^2^/h, with a lag time of 0.83 h. The permeation parameters obtained from nasal mucosa were comparable with those obtained using a semi-permeable membrane with a flux of 72.5 µg/cm^2^/h with a lag time of 0.96 h (Table 6).

### 3.8. Stability

**Figure 8:**
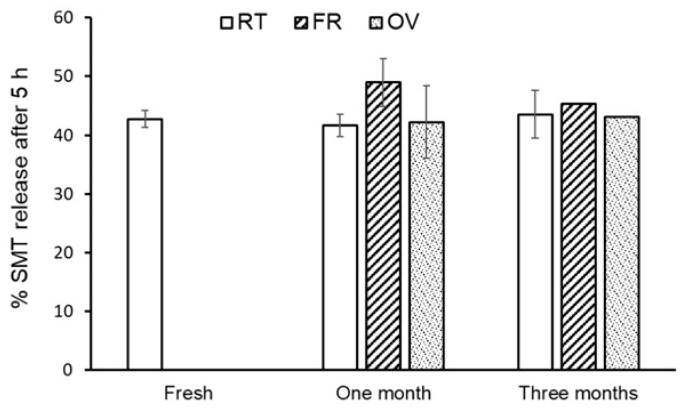
Stability release study of sumatriptan from the in situ nasal gel optimized formula after 5 h at different conditions.

The stability of the optimized formula was monitored for 1 and 3 months at room temperature, in the oven (40 °C), and in the fridge. The characterization included gelation temperature, pH, viscosity, drug content, and drug release. No change was measured in the pH, ranging from 5.5 to 5.6. After a visual inspection of the gel, it was found that the gel formulation stored at room temperature and in the fridge kept their its character, while those stored in the oven turned yellowish. Figure 8 shows the percent of SMT released in different storage conditions. The released data showed no change in the amount of SMT released compared with the freshly prepared formulation. The degradation rate constants were $4.6\times 10^{-4}$, $9.6\times 10^{-4}$, and $1.5\times 10^{-3}$ at 4, 25, and 40 °C, respectively [31].

## 4. Conclusions

In this study, a thermosensitive mucoadhesive in situ nasal gel for SMT was successfully developed utilizing a systematic approach grounded in 3D response surface methodology. Here, nine formulations were created, using PLU F-127 and chitosan concentrations as the independent factors. Comprehensive assessments of pH, clarity, drug content, gelation properties, mucoadhesive strength, viscosity, and release kinetics were conducted. The optimized formulation yielded a clear gel with a rapid gelation time of less than 1 min at a gelation temperature of 33 °C. Furthermore, the gel showed a 40% drug release and a 50% permeation after 5 h. Importantly, the retention time in the nasal cavity was enhanced due to the mucoadhesive property of the gel and its effect to reduce the mucociliary clearance, hence potentially improving the SMT absorption and efficacy. The stability of the optimized gel formulation, evaluated under accelerated conditions, further supports its viability for therapeutic use. Overall, the findings indicate that this formulation holds promise for enhancing the delivery and effectiveness of SMT in clinical applications.

## Figures and Tables

**Table 1:** Composition of SMT *in situ* gel formulations.

Formula	F1	F2	F3	F4	F5	F6	F7	F8	F9
SMT	5 mg/0.1 mL								
PLU F127 (%)	13.5	18	22.5	13.5	18	22.5	13.5	18	22.5
HMW chitosan (%)	0.01	0.01	0.01	0.03	0.03	0.03	0.05	0.05	0.05

**Table 2:** Characterization of SMT *in situ* gel prepared with HMW chitosan.

	Gelation Temperature (°C)	Gelation Time (s)	Gelation Strength (s)	Mucoadhesive Strength (Dyne/cm^2^)	% Release After 5 h
F1	>70	>300	0.003 ± 1.15 × 10^4^	1295	57.920 ± 10.862
F2	31.667 ± 0.153	31 ± 1	4.500 ± 0.5	1750	52.480 ± 2.351
F3	24 ± 0.100	32 ± 1	94 ± 1	1295	37.508 ± 1.346
F4	>70	>300	0.003 ± 1.15 × 10^4^	1295	65.350 ± 5.035
F5	31.800 ± 0.100	31 ± 1	6 ± 1	2100	50.341 ± 6.454
F6	23.900 ± 0.100	13 ± 1	124 ± 1	2100	41.107 ± 10.318
F7	>70	>300	0.003 ± 1.15 × 10^4^	1505	49.145 ± 2.497
F8	31.700 ± 0.200	34 ± 1	3 ± 1	2170	49.292 ± 2.708
F9	24.033 ± 0.153	17 ± 1	78 ± 1	2100	47.731 ± 4.530

**Table 3:** Rheological pattern for SMT in situ gel.

Formulations	Eta (mPa∙s)			
RPM	20	50	70	100
F1	-	-	-	4.449 ± 0.665
F2	33.418 ± 2.018	41.782 ± 0.168	44.406 ± 0.100	45.788 ± 0.387
F3	153.936 ± 17.241	142.250 ± 8.603	139.474 ± 4.659	138.326 ± 4.700
F4	-	-	3.772 ± 0	3.665 ± 1.149
F5	23.390 ± 0.142	30.778 ± 1.700	33.274 ± 1.092	35.25 ± 0.796
F6	98.562 ± 0.836	105.411 ± 4.976	106.553 ± 6.346	106.898 ± 7.115
F7	-	-	4.267 ± 0.359	5.7615 ± 0.128
F8	19.202 ± 3.112	26.767 ± 3.120	29.332 ± 3.033	31.045 ± 3.329
F9	114.973 ± 18.975	125.417 ± 15.073	126.164 ± 15.474	126.455 ± 15.605

**Table 4:** Release kinetics model for SMT from different *in situ* gel formulations.

	Zero-Order Model		First-Order Model		Higuchi Diffusion Model		Korsmeyer–Peppas Model	
	R	Slope	R	Slope	R	Slope	R	n
F1	0.954	0.182	−0.977	−0.001	0.980	4.003	0.936	0.766
F2	0.996	0.173	−0.999	−0.001	0.993	3.682	0.998	0.918
F3	0.999	0.121	−0.998	−0.001	0.987	2.556	0.999	0.923
F4	0.996	0.214	−0.999	−0.002	0.996	4.579	0.999	0.860
F5	0.998	0.165	−0.998	−0.001	0.990	3.491	0.999	0.925
F6	0.997	0.124	−0.994	−0.001	0.987	2.627	0.998	0.789
F7	0.994	0.166	−0.999	−0.001	0.996	3.561	0.996	0.949
F8	0.999	0.163	−0.998	−0.001	0.989	3.458	0.999	0.970
F9	0.999	0.156	−0.993	−0.001	0.9787	3.257	0.997	0.950

**Table 5:** The selected optimized formula predicted and observed results.

Formulation Composition	Parameters	Predicted Value	Observed Value
PLU F-127 (17.972%) Chitosan (0.03%)	Gelation temperature (°C)	31.842	33.03 ± 0.115
	Gelation time (s)	30	53 ± 1
	Gelation strength (s)	13.179	5.6 ± 0.454
	Mucoadhesive strength (dyne/cm^2^)	2099.267	1423 ± 0
	% released after 5 h	54.880	42.748 ± 1.436
	p^H^		5.5
	Clarity		Clear
	Drug content %		102.319 ± 0.764

**Table 6:** Permeation parameters of SMT from the *in situ* gel through the semipermeable membrane and fresh nasal mucosa.

Membrane Type	Cumulative Amount Permeated (µg/cm^2^) After 5 h	Flux (µg/cm^2^/h)	Lag Time (h)
Nasal mucosa	3920.165 ± 171.330	90.870 ± 4.790	0.83 ± 0.003
Semipermeable membrane	3080.300 ± 4.990	75.553 ± 4.270	0.895 ± 0.240

## Data Availability

The original contributions presented in this study are included in the article/Supplementary Materials. Further inquiries can be directed to the corresponding author.

## Supplementary Materials

The following supporting information can be downloaded at: https://www.mdpi.com/article/10.3390/polym16233422/s1.
